# Vitamin E Metabolic Effects and Genetic Variants: A Challenge for Precision Nutrition in Obesity and Associated Disturbances

**DOI:** 10.3390/nu10121919

**Published:** 2018-12-04

**Authors:** Sebastià Galmés, Francisca Serra, Andreu Palou

**Affiliations:** 1 Laboratory of Molecular Biology, Nutrition and Biotechnology, NUO Group, Universitat de les Illes Balears, 07122 Palma, Spain; s.galmes@uib.cat (S.G.); andreu.palou@uib.es (A.P.); 2 CIBER de Fisiopatología de la Obesidad y Nutrición (CIBEROBN), 28029 Madrid, Spain; 3 Institut d’Investigació Sanitària Illes Balears (IdISBa), 07120 Palma, Spain

**Keywords:** genetic variant, tocopherol, nutrigenetics, metabolic syndrome, obesity, vitamin E

## Abstract

Vitamin E (VE) has a recognized leading role as a contributor to the protection of cell constituents from oxidative damage. However, evidence suggests that the health benefits of VE go far beyond that of an antioxidant acting in lipophilic environments. In humans, VE is channeled toward pathways dealing with lipoproteins and cholesterol, underlining its relevance in lipid handling and metabolism. In this context, both VE intake and status may be relevant in physiopathological conditions associated with disturbances in lipid metabolism or concomitant with oxidative stress, such as obesity. However, dietary reference values for VE in obese populations have not yet been defined, and VE supplementation trials show contradictory results. Therefore, a better understanding of the role of genetic variants in genes involved in VE metabolism may be crucial to exert dietary recommendations with a higher degree of precision. In particular, genetic variability should be taken into account in targets concerning VE bioavailability per se or concomitant with impaired lipoprotein transport. Genetic variants associated with impaired VE liver balance, and the handling/resolution of oxidative stress might also be relevant, but the core information that exists at present is insufficient to deliver precise recommendations.

## 1. Introduction

The term “Vitamin E” (VE) refers to a set of lipid-soluble compounds that includes both tocopherols and tocotrienols. Tocopherols represent the best part of VE consumed around the world, with γ-tocopherol being the most consumed by Americans [1], and α-tocopherol being the most represented in European diets [2]. In mammals, VE plays an important role as an antioxidant due to its capacity to bind free radicals. Mechanistically, this takes place if the hydroxyl group of the chromanol ring is not esterified, for example to a phosphoric acid, or is hidden by protein binding. In addition, tocopherols have a lipophilic tail that is able to interact with cellular lipids and other molecules, including DNA, protecting them from oxidation or peroxidation damage. In addition, severe α-tocopherol deficiency causes important neuronal disorders, such as ataxia, and oxidative-based disorders, such as cardiovascular disease, cancer, or cataracts [3]. In fact, European food products that are considered important sources of VE are able to bear the claim “Vitamin E contributes to the protection of cell constituents from oxidative damage”, as this role has been recognized by the European Food Safety Authority (EFSA) [4]. However, VE benefits to health go beyond its lipophilic antioxidant potential in cellular structures. Different forms of VE and its derivatives may act as modulators of enzymes mainly involved in signal transduction, affect gene expression (e.g., redox-regulated), have immunomodulatory properties, and play a relevant role in degenerative diseases [5]. Recently, several reviews have addressed a number of new potential roles of VE, which is still not totally understood or characterized [5,6,7,8,9,10,11]. Furthermore, some studies have outlined the relevance that VE and derivatives may have in obesity, cardiovascular disease (CAD), and metabolic-related diseases. Critical pathways involved in metabolic syndrome are under the influence of VE, and evidence suggests that in specific cases, adequate supplementation would be an appropriate strategy to help in treatment of the prevention of obesity and its associated comorbidities [12,13]. However, dietary reference values for VE in obese populations have not yet been defined, and clinical trials show contradictory results with VE supplementation [5]. In fact, insufficient data on biomarkers and methodological uncertainties to estimate VE intake, together with inconsistent effects on health, have led to the proposal of adequate intakes (AI) of α-tocopherol, which have been based on observed intakes in a general and apparently non-VE deficient population [14]. The causes can be multiple and interconnected: serum concentrations of VE are increasing in an age-dependent manner, and are highly influenced by different blood lipids. No good methods to control for this confounding effect have been effective. Furthermore, very heterogeneous α-tocopherol plasma levels are found in the general population (range 19.9–34.2 µmol/L); even within one country, values may range from 5 µmol/L to 35 µmol/L, and this is accompanied by a significant percentage of the population (79%) not reaching the proposed desirable concentration of 30 µmol/L, at which beneficial effects may occur [15], making it even more difficult to address the impact of VE homeostasis when suboptimal levels are the ‘standard’ situation in the population.

In addition, there is no clear evidence that obese individuals have different VE levels in plasma than healthy people, which is contrary to the association of low vitamin D levels with obese individuals [16]. In fact, obese individuals may show either low [17] or high [18] levels of plasma VE in comparison with normal weight subjects in different studies. People with metabolic syndrome (MetS) may show half of the plasma VE found in the reference group (i.e., 12.5 µmol/L versus 25.5 µmol/L), despite a similar intake of VE (8.85 mg versus 9.33 mg of α-tocopherol [18]. Regardless of the expected benefits from VE consumption regarding CAD and even cancer, some supplementation trials did not succeed in showing them [19]. Indeed, the beneficial effects of VE have been recently revised, highlighting some important controversy regarding CAD and the mortality effects of VE supplementation [5]. A meta-analysis concluded that VE supplementation (400–800 IU) decreased the risk of suffering both non-fatal and fatal myocardial infarction, while this preventive effect did not appear using other antioxidants [20]. However, concerning stroke relative risk, supplementation with VE (50–800 IU) did not seem to affect the total stroke risk, but did increase the risk of hemorrhagic stroke by 22% [21]. In addition, the uptake of supplemented VE varies widely among individuals but, after only one year of treatment, the response seems to be homogenized [22]. Therefore, polymorphisms in the key genes dealing with the physiological management of VE, or in genes coding for proteins that execute its bioactive properties, emerge as a feasible explanation for the VE variability observed in these trials. In fact, genetic variants associated with circulating VE levels [23], bioavailability, absorption, and metabolism [24,25,26] have been identified during the last few years [27]. Recently, a set of 28 single nucleotide proteins (SNPs), concentrated on 11 genes, has been considered a useful tool in explaining a significant part (82%) of the interindividual variability of VE bioavailability in healthy humans [26].

The emerging concept of “precision nutrition” is based on new knowledge, which is mainly derived from –omic biomarkers describing specific characteristics of individuals, and aims to define more effective, personalized nutritional guidelines [28]. This type of approach takes into account genetic variants that modulate the effective benefits of VE. Thus, specific requirements can be achieved by means of more accurate intake, adjusted to the individual’s bioavailability. However, only a few studies have carried out research on the influence of relevant genetic variants on the effects of VE and its metabolism, and so far, a holistic perspective is still missing. Thus, the objective of this review is to gain a better understanding of the nutrigenetic influence of VE, by specifically analyzing those that could be particularly relevant in the prevention or treatment of obesity and its associated complications.

## 2. Vitamin E Metabolism: An Overview

VE and its derivatives are present in meals and appear mixed with other lipophilic molecules just before being absorbed from the food matrix by the enterocytes. The intestinal uptake of VE involves three main proteins [29], which are neither substrate-specific nor tissue-specific, as they are able to import other cargo proteins and are shared by other organs (such as the liver) [30]. Niemann–Pick C1 (NPC1), scavenger receptor class B type 1 (SR-B1), and cluster determinant 36 (CD36) are involved in VE uptake on the apical side of the enterocyte; they share common uptake pathways and are clearly associated with the transmembrane transport of cholesterol and other lipophilic components such as VE [30,31,32,33,34]. VE enterocyte internal carriers are still not clear, but NPC1 and NPC2 may play such a role [35]. Then, when VE reaches the basolateral side of the enterocyte, most of the VE is secreted in the chylomicron fraction [29]. However, a low percentage of VE is incorporated and secreted into intestinal high-density lipoprotein (HDL) by ABCA1 [36] with the contribution of Apo-AI, which mediates the α-tocopherol efflux from intestine cells to HDL [37]. In fact, no specific plasma transport proteins for α-tocopherol have been described, and VE transport in blood shares the cholesterol and lipoprotein pathways [38]. Then, the flux of lipoproteins toward the liver facilitates hepatic VE delivery. For instance, most of the VE remains in the chylomicron particle during triglyceride lipolysis by lipoprotein lipase (LPL) and, when remnant chylomicrons are directed to the liver, the ApoB receptor facilitates VE internalization. Then, VE may also be imported into the liver via the low-density lipoprotein (LDL) receptor (LDLR), lipoprotein-related proteins (LRP), and SR-B1. In the same way, VE associated with HDL particles is imported into the liver owing to SR-B1 [39]. Furthermore, extrahepatic tissues can receive VE from chylomicrons, via very low-density lipoprotein (VLDL)/LDL uptake through specific receptors or from the LPL product [11]. Concerning hepatic VE distribution, VLDL leaves the liver enriched in α-tocopherol (approximately 65 molecules per particle [40]); its subsequent conversion to LDL gives α-tocopherol-enriched LDL particles (approximately eight to 12 molecules per particle), whereas HDL contains less than one α-tocopherol per particle [40]. Generally, it is believed that LDL synthesized by the liver provides other tissues with VE, while extrahepatic tissues can also provide VE to the liver by reverse cholesterol transport [41].

VE is not transformed into bioactive forms in enterocytes or along systemic transport. Then, in the liver, α-tocopherol is specifically selected and bound by α-TTP (α-tocopherol transfer protein) protecting its side-chain oxidation and facilitating VE transfer to liver nascent lipoproteins. The remaining forms of VE may undergo -hydroxylation by cytochrome P450-mediated metabolism [42,43]. The end products of VE catabolism are a group of metabolites—short-chain and long-chain derivatives—that have traditionally been considered excretion products. However, some of those long-forms have recently been found in plasma, with their biological activity assayed in in vitro systems, and are now considered a new class of regulatory metabolites (for further details, see Galli et al. (2017) and Schmölz et al. (2016) [5,11]). For more detailed reviews concerning pathways involving VE and its metabolism, we suggest looking at these authors Takada et. al. (2010), Reboul et al. (2011), Kaempf-Rotzoll (2003) and Brigelius-Flohe (1999) [36,39,44,45].

## 3. Influence of Genetic Variants on Transport, Management, and Effects of VE

The main focus of this review is to gain a better understanding of genetic variants that could mediate VE metabolic effects in order to define guidelines that are able to support more precise nutrition for obesity and related diseases. Strategies considering VE supplementation should take into account genetic variability in targets concerning VE bioavailability, catabolism, and function. In this context, four main areas of interest have been identified: (a) VE bioavailability per se, (b) VE bioavailability concomitant with impaired lipoprotein transport; (c) VE bioavailability associated with impaired VE liver storage or catabolism balance; and (d) VE bioavailability compromising the resolution of oxidative stress.

### 3.1. Impaired Bioavailability of VE

Polymorphisms in genes coding proteins involved in the absorption of VE in the enterocyte could influence the ability to acquire VE and thus modulate its bioavailability [25]. Nutrigenetics may provide more focused specifications concerning the amount of VE that an individual should ingest so as to reach the necessary dose. Thus, genotypes with a lower capacity to acquire VE from the diet would require greater dietary presence or taking supplements. That main proteins that are involved in the transport of tocopherol are CD36, SR-B1, NPC1, and ATP-binding cassette (ABC) transporters [36].

Cluster determinant 36 (CD36) is a membrane glycoprotein that is involved in a wide range of functions in different cell types, with a leading role in the coordination of cellular events involved in the uptake and processing of fatty acids (FA). Therefore, CD36 expressed on the apical side of enterocytes of the proximal intestine interacts with dietary FA released from the digestion of triglycerides, facilitating VE uptake and chylomicron assembly [46]. CD36 is also involved in the uptake of long-chain free FA to drive beta-oxidation in myocytes [47] or for their storage in adipocytes [48]. Moreover, CD36 recognizes a number of lipid compounds, including cholesterol [49] and carotenes [50]; it binds native and oxidized lipoproteins, thereby promoting cellular adhesion and internalization of oxidized LDL (ox-LDL) in monocytes [51,52,53], and also contributes directly or indirectly to the transport [34] and internalization of VE [54]. Another molecular characteristic of CD36 is that it seems to be a key element in the detection of the taste of fat, contributing to both lingual and intestinal fat sensing [55].

Thus, genetic variants related to the concentration of CD36 have been associated with differences in creaminess perception regardless of fat content, increased body weight, and waist circumference [56]. Furthermore, the expression of CD36 is reduced by α-tocopherol, while VE reduces the uptake of ox-LDL by inhibiting *CD36* expression, altogether suggesting the involvement of CD36 in the metabolism of VE [57,58,59]. So far, there are two relevant SNPs located on CD36 gene that have been related to plasma α-tocopherol concentrations. Both rs1761667 and rs1527479 are found in high linkage disequilibrium [60]; therefore, we analyze the role of the former, as it has been the most characterized in this aspect. 

Carrying the A allele of the polymorphism located in the intron region of the *CD36* gene (rs1761667) has been associated with a reduced expression of the CD36 transcript, as well as of total and surface protein in monocytes compared with allele G. In addition, the A allele has been associated with a reduced sensitivity to fatty taste, together with an increased perception of creaminess and greater preference for fats added to foods [61]. It has been reported that CD36 variants that reduce protein expression appear to promote a protective profile concerning circulating lipoproteins [60]. In this context, the Healthy Lifestyle in Europe by Nutrition in Adolescence (HELENA) study identified that homozygous individuals for the G allele had lower plasma α-tocopherol concentration (3%), but this relationship did not attain statistical significance when corrected for multiple testing [60]. Furthermore, although most of the CD36 polymorphisms are not strongly associated with obesity, they clearly contribute to interindividual variability in plasma lipid and lipoprotein profiles, therefore influencing cardiovascular risk [62]. As lower CD36 expression seems to be metabolically protective, the promotion of high VE intake in appropriate subjects may be a dietary strategy to counteract excess CD36 protein, aiming toward a healthier lipid profile. 

Scavenger receptor class B member 1 (SR-B1) is coded by *SCARB1* gene, and is a multi-ligand membrane receptor extensively expressed in many mammalian cell types, including enterocytes, myocytes, endothelial cells, adipocytes, and macrophages. To date, SR-B1 is the only known bidirectional integral membrane protein in the apical site of enterocytes [32]. The protein acts as a plasma membrane receptor for high-density lipoprotein cholesterol (HDL) and mediates cholesterol transfer to and from HDL. Thus, SR-B1 deficiency results in hypercholesterolemia [63,64,65]. Concerning VE, SR-B1 is involved in the uptake of the main forms of VE from the diet; it participates in its transport from the basolateral site of enterocytes to the blood, the uptake of VE vitamers–HDL complexes by different acceptor tissues, and the excretion of α-tocopherol with biliary secretion [36]. Liver uptake of VE is carried out by SR-B1, whereas in extrahepatic tissues, VE is internalized and mixed with triglycerides by the action of lipoprotein lipase (LPL) [66,67]. Four SNPs located on *SCARB1* exons and one located on an intron region are known to influence VE metabolism. Furthermore, an interaction between gender and the presence of genetic variants in the SCARB1 gene has been shown to influence plasma tocopherol concentrations [68].

The minor allele (T) of the missense variant Gly2Ser (rs4238001) has been associated with lower HDL-cholesterol and LDL-cholesterol in type 2 diabetics in the Framingham Heart Study [69]. Furthermore, the T allele has been associated with higher SR-B1 degradation and lower protein levels [64]; a recent meta-analysis across large race and ethnic population groups concluded that this variation is also associated with greater risk of coronary heart disease [70]. Rs5888, which is also known as A350A, is a synonymous variation located on exon 8 of Scavenger receptor class B type 1 gene (SCARB1). The exchange of the minor variant (C) for T has been related to splicing activity [71]. Dyslipidemia, coronary heart disease, and related disturbances have been found to be associated with the presence of this polymorphism in a gender and age-dependent manner, but there are no conclusive data regarding its precise role [72]. However, the consequences of this gene variation remain unclear: CT carriers were associated with an atherosclerosis-protective effect in a Lithuania population-based study [73], while in another study, it was the heterozygous carriers (CT) that had an increased risk of age-related macular degeneration in French and North American populations, due to a potential impaired function of SR-B1 as a transporter of functional lipophilic compounds such as cholesterol, lutein, and VE [74].

Therefore, the poor bioavailability of antioxidants in particularly dependent tissues would make the carriers of the minor genetic allele more prone to showing a pro-inflammatory profile or a deficit in redox homeostasis. In this way, it is remarkable that TT carriers had lower α-tocopherol levels in plasma than CC and CT carriers (all male subjects), with the latter having higher levels (+16.4% more than TT) [68]. Finally, the presence of the variant allele of the polymorphism located on the intron region of SCARB1 gene (rs11057830) was clearly related to lower plasma α-tocopherol in a genome-wide association study, and has been further confirmed in the analysis of a supplementation trial [23]. Therefore, a body of evidence clearly links the lower expression or functionality of SR-B1 to disturbances in lipid metabolism, including postprandial triglyceride response and the handling of lipoproteins, as would be expected due to its main role as an HDL transporter. These situations can be worsened in the case of obesity, particularly if lower levels of plasma VE are found. Although more research is needed in order to discern whether greater VE intake would accompany higher plasma levels of tocopherol, this could be a dietary strategy aiming to counteract some of the effects associated with impaired SR-B1 function. 

Niemann–Pick disease type C1 (NPC1) is a membrane protein that is highly expressed in the intestine, macrophages, and liver, and plays a crucial role in the absorption and movement of lipophilic compounds across cell membranes [75]. A number of mutations in the *NPC1* gene have been associated with lysosomal storage disorders [76], and concerning obesity, a Genome Wide Association (GWA) study found a role for the non-synonymous variant rs1805081 (His215Arg) in early-onset and morbid adult obesity in the European population [77], and follow-up studies have shown the involvement of common genetic variants in adult-onset obesity, body fat mass, and type 2 diabetes [78]. Furthermore, NPC1 is the pharmacological target of ezetimibe, which is an inhibitor of cholesterol endocytosis that is widely used for treatment of this dyslipidemia [79]; its inhibition of α-tocopherol absorption [80] suggests the involvement of NPC1 in dietary VE uptake. Mechanistically, α-tocopherol is known to bind competitively with cholesterol at the N-terminal domain of NPC1, which is essential for the endocytosis of both compounds into the cell [75]. In vitro experiments using Caco-2 cells transfected with plasmids overexpressing the genetic non-synonymous variants found in low cholesterol absorbers, showed less transport activity of both cholesterol and α-tocopherol in comparison with wild-type cells [81]. Experimental evidence supports that the following NPC1 polymorphisms are able to reveal diminished transport activity (21–63%) and, as such, may influence bioavailability of VE: Ala395Val (rs62001882), Gly402Ser (rs141973731), Arg417Trp (rs139659653), and Gly434Arg (rs114375162) [81].

The ATP-binding cassette transporter A1 (ABCA1) is a 220-kDa member of the ABCA transporter family that is highly expressed in liver, glands, intestine, and macrophages. It carries out the hepatic outflow of cholesterol and phospholipids onto apolipoprotein A-1 (Apo-AI), forming nascent high-density lipoprotein (HDL) particles [82]. In intestine, ABCA1 located in the basolateral membrane of enterocytes, is crucial for absorption, transport, and secretion into the circulation of cholesterol, phospholipids, and other lipophilic compounds, mainly packaging them into HDL particles [83]. Evidence has been collected from Tangier disease’s patients, who suffer from a deficiency of the *ABCA1* gene and show an absence of serum HDL, hypertriglyceridemia, and reduction in LDL serum levels. Impaired cholesterol and lipid transport functions result in the ectopic accumulation of cholesteryl esters; consequently, these patients have a higher incidence of coronary heart and artery disease [84,85]. Data indicate that ABCA1 is also responsible for the secretion of VE into portal blood in the form of intestinal HDL [36].

Apart from ABCA1 as a key player in HDL formation, ABCG1 is another member of the ABC family, which is responsible for transfering cholesterol from macrophages or vascular endothelial cells to mature HDL particles, which represent a large fraction of overall plasma HDL. Therefore, ABCG1 is responsible for the reverse cholesterol pathway, which is critical for lipid clearance and the transfer of excess cholesterol from peripheral tissues back to the liver [85].

These two transporters participate in vitamin E efflux from cells. The relationship between VE levels and ABCA1 was confirmed in mice with targeted *ABCA1* inactivation, which showed decreased levels of HDL as well as VE in plasma [86]. Concerning ABCG1, it has a general impact on VE repartition within the organism; therefore, its repression induces a decreased efflux from cells to HDL, leading to VE accumulation in tissues as seen in knockout animals [87].

Taking into account the role of both ABC family transporters, genetic variants would be expected to have a relevant influence on cholesterol transport and lipid metabolism, and therefore on VE bioavailability and distribution. Three genetic variants located near the ABCA1 gene (rs4149268, rs3890182, and rs1883025) have been associated with HDL concentrations in different studies [88,89,90,91], although no gene–vitamin E significant interaction with HDL levels has been found concerning any of these variants [92]. Whereas the variants rs11789603, rs2274873, rs4149314, and rs4149297, when analyzed for their potential role in interindividual variability in α-tocopherol bioavailability, have been associated with lower VE levels after an α-tocopherol rich meal [26], no information regarding cholesterol transport and lipoprotein metabolism has been addressed in parallel. Therefore, in the case that lower VE bioavailability is demonstrated in relation to the presence of specific genetic variants in *ABCA1* or *ABCG1* genes, and involves a lower transcription rate, and/or lower transporter concentrations, it remains inconclusive whether VE supplementation would be a reliable strategy. Evidence points to greater VE consumption predisposing to the mRNA suppression of ABC transporters, and this profile has been associated with higher cardiovascular risk, at least in animal models [87].

### 3.2. High-Plasma Vitamin E Associated with Impaired Lipoprotein Transport

As outlined in the introduction, the handling and transport of VE is highly complex, as it is mediated by a number of proteins shared within metabolic pathways dealing with other lipophilic compounds. Despite the existence of relatively redundant mechanisms, any genetic variation affecting lipid systemic transport performance is also a potential target for VE bioavailability disturbance. Plasma α-tocopherol has been used as a proxy to assess VE status, although an association between dietary intake and plasma α-tocopherol concentration has not been found consistently [14]. In the previous section, impaired VE bioavailability per se was the main cause of low VE plasma levels, and in most cases, higher VE intake would be expected to compensate for the dysfunction; this is unlike the variants considered in this section, which are mainly related to lipoprotein malfunctioning. Most of the risk alleles of the gene variants described in the apolipoproteins that are relevant to VE management are associated with altered levels of blood lipids and, consequently, a greater amount of VE in plasma, due to decreased VE availability in the liver and other tissues. At this point, recommendations of a higher VE intake to cope with tissue deficiency remains a research question to be specifically addressed.

The relationship between VE circulation and lipoprotein metabolism is intimately related, and is well described elsewhere [93,94]. Briefly, after intestinal absorption, VE forms are delivered to the lymph packaged into chylomicrons to be later secreted to the blood circulation. Once there, VE could be incorporated into tissues by lipoprotein lipase (LPL) action or reach the liver to be stored or put back into circulation as part of the lipoproteins of hepatic origin. In this context, apolipoprotein synthesis and metabolism play a crucial role in tocopherol and tocotrienol transport because, as commented, VE and its derivatives can be actively exchanged between the different circulating lipoproteins. In fact, positive correlations between VE isoforms and different circulating apolipoproteins have been observed, which is at the basis of a proposed plasma proteome to characterize VE deficiency in children [95]. As the main apolipoproteins involved in α-tocopherol circulation, it is worth considering Apo-AI and Apo-AV, which belong to a gene cluster, Apo-AII, Apo-E, and Apo-B. The gene encoding for Apo-AI is clustered on chromosome 11 with other apolipoproteins (Apo-AIV, Apo-AV, and Apo-CIII) [96]. Apo-AI is the main protein component of nascent and mature HDL; it is synthesized in the liver (80%) and small intestine (10%), and as a cofactor for lecithin cholesterol acyltransferase, it supports cholesterol efflux from tissues [97].

Apo-AI allows the movement of VE from the enterocyte into the bloodstream, but it is also present in mature HDL particles. Therefore, it favors the reverse transport of cholesterol and, in turn, of tocopherol from the tissues to the liver [98]. A number of genetic variants have been involved in apolipoprotein metabolism, and hence in VE nutritional status. The presence of the A allele from rs670 has been associated with an in vitro higher gene transcription rate of Apo-AI, as well as with higher levels of HDLc and Apo-AI in plasma [99]. Consequently, the presence of allele A is associated with higher levels of HDL, and lower risks of visceral obesity, diabetes, and hypertriglyceridemia [100].

Apo-AV constitutes a minor apolipoprotein that is almost exclusively expressed in liver, and is found in plasma at low concentrations as well as intracellularly in association with lipid droplets, where it seems to participate in intracellular triglyceride regulation. Apo-AV is a potent regulator of triglyceridemia, and human deficiency leads to hypertriglyceridemia. This apolipoprotein participates in the assembly of VLDL, and is found in HDL and chylomicrons, but not in LDL [101]. Its main role has been characterized with regard to its interaction with LPL, promoting chylomicron clearance, and hence decreasing circulating triglycerides [102]. Obese and diabetic individuals have lower plasma levels of Apo-AV in comparison with healthy subjects [103,104], which may be because insulin is a negative regulator of the *APOA5* gene. Furthermore, a number of studies provide evidence for the interaction between genetic variants associated with APOA5 in the modulation of lipid metabolism and an increased risk of obesity and metabolic syndrome (for reviews, see Guardiola et al. (2017) and Girona et al. (2018) [105,106]). The most studied SNP located on the *APOA5* promotor is probably 1131T>C (rs662799), in which the minor variant (C) has been associated with lower Apo-AV plasma concentration, which correlates with dyslipidemia. Minor allele carriers show remodeling of the lipoprotein profile toward atherogenic dyslipidemia, which is characterized by large VLDL, and small LDL and HDL [105]. Concerning the potential modulation of VE by *APOA5* in diabetic patients carrying the C variant of rs662799, higher plasma triglycerides are shown in combination with higher plasma VE, although this is not associated with improved antioxidant parameters [107]; similar results have been found in healthy school children [108]. Another relevant SNP on *APOA5* is rs3135506, which codifies the exchange of a serine for a tryptophan (S19W or 56C>G). The G allele is very rare worldwide, and has been associated with a great prevalence for metabolic syndrome, as it is accompanied by a higher prevalence of plasma hypertriglyceridemia [109] and greater α-tocopherol [107,110]. In addition, GWA studies have provided details about potential variants close to *APOA5*, which also correlate with higher levels of plasma α-tocopherol. However, they are able to explain only a small fraction (1.7% of the unexplained variance) of the interindividual variance on α-tocopherol concentration [23,110]. 

Apo-AII is the second most abundant protein in HDL [111]; it takes part in mature HDL particles, and participates in the transfer of lipophilic substances between HDL and tissues, such as phospholipids [112] or α-tocopherol [113]. The presence of the T/T genotype of rs5082 has been associated with postprandial hypertriglyceridemia response and greater cardiovascular risk compared with individuals carrying C/T or C/C allele combinations [114].

Apo-E is a multifunctional protein that mediates the binding, internalization, and catabolism of lipoprotein particles. Apo-E acts as a ligand for lipoprotein receptors and mediates clearance from the plasma of triglyceride-rich lipoproteins and remnant VLDL and chylomicron [115]. Three main APOE alleles have been identified: ApoE-2 (Cys112, Cys158), ApoE-3 (Cys112, Arg158), and ApoE-4 (Arg112, Arg158); the small differences derived from these two amino acid positions have relevant consequences in the structure and function of Apo-E, thereby modulating the prognosis and progression of cardiovascular disease. The most common isoform (3) is present in 40–90% of the population, and is considered the ‘neutral’ Apo-E genotype [116]. In ApoE-2, the receptor-binding region is inactivated, which results in the delayed clearance of hepatic and intestinal remnant lipoproteins, hyperlipoproteinemia, and seems to be associated with a mild form of atherosclerosis progression. Whereas the structural change defined in ApoE-4 reshapes the lipid-binding site and then targets large triglyceride-rich VLDL rather than small phospholipid-rich HDL, individuals with ε4 allele show higher levels of LDL cholesterol, which favors the induction of atherosclerotic lesions [117]. Some studies have estimated that carriers of the ApoE-ԑ4 allele may have a 42% higher risk of cardiovascular disease than carriers of the ApoE-ԑ3/ԑ3 genotype; they also have a greater risk of Alzheimer’s disease, and in general the genotype is negatively associated with longevity [118,119,120]. Although discrepant results have been obtained, the ApoE genotype may be expected to influence VE status. In humans, the lowest plasma VE concentrations were found in Apo-ԑ2/ԑ2, whereas the presence of the ԑ4 allele was accompanied by the highest plasma levels. A range of 14.2 µmol/L was observed between ԑ2/ԑ2 and ԑ4/ԑ2 genetic groups [68], which could be indicative of lower retention in peripheral tissues. It has been proposed that the lower VE tissue levels observed with the Apo-E4 genotype may be due to different reasons, such as: (a) decreased catabolism of LDL and VE retention; (b) decreased expression of lipoprotein receptors, which will delay the cellular uptake of vitamin E in Apo-E4 carriers; (c) activated intracellular degradation of tocopherols in the Apo-E4 genotype, contributing to lower VE in peripheral tissues; and (d) lower levels of SR-B1 accompanying impaired cellular VE delivery through this [121]. ApoE-ԑ4 carriers might have an increased demand for VE in order to counteract both the reduced uptake into extrahepatic tissues and the higher degradation rate [122]. Furthermore, genetic variants may contribute to exacerbate the impact of ApoE alleles, as happens with rs449647 (491A>T), which is located in the promoter of the gene, and results in 63% decrease in its activity [123,124].

Concerning Apo-B, the Apo-B gene codifies a single transcript that generates a larger protein, Apo-B100, which is found in lipoproteins originating from the liver, such as Very Low-Density Lipoprotein (VLDL), Intermediate-Density Lipoprotein (IDL) and Low-Density Lipoprotein (LDL), and a shorter isoform, exclusively formed in intestine, and essential for chylomicron assembly (Apo-B48). Apo-B proteins are recognized by the Apo-B receptor and are crucial for liver HDL (and VE) uptake mediated by the SR-B1 protein.

Concerning Apo-B variants, in three GWAs, rs693 minor allele (T) was associated with a greater risk of elevated total cholesterol [125] and LDL-cholesterol levels in blood [88,91,126,127], indicating a strong influence of this variant on the risk of dyslipidemia. A lifestyle intervention based on calorie restriction and physical activity proved to be useful for the carriers of the most frequent allele of rs693, but not for carriers of the minor allele, in an attempt to improve cholesterol profile [127]. An interaction between rs693 with serum LDL cholesterol and VE has been reported in non-Hispanic whites and Mexican-American populations [92]. Moreover, in an attempt to explain the heterogeneous response to VE bioavailability (estimated coefficient of variation of 81%), the Apo-B variants rs1713222 (G allele), rs4643493 (T allele), and rs1042031 (T allele) have been associated with higher plasma α-tocopherol in chylomicrons after the consumption of VE-rich meal food [26].

Some evidence points out that the risk alleles for apolipoproteins related with VE metabolism lead to a longer lapse of circulating lipids [128] and predispose to higher levels of blood lipids. They are also associated with a reduced ability to incorporate lipophilic compounds into cells, since VE is highly hydrophobic, and cannot adequately access inside cells to carry out its functions, including antioxidant activity. In this scenario, obese people are especially vulnerable to suffering dyslipidemia and chronic low-grade inflammation [129], and the presence of apolipoprotein risk variants would impair taking advantage of the properties of vitamin E, even when following current dietary recommendations.

Data concerning apolipoproteins and VE interactions are scarce, and in most cases do not come from studies aiming to specifically assess the influence of VE on the antioxidant profile or on lipid handling in relation with the presence of genetic variants. Although more focused research is needed, it could be anticipated that a main nutritional objective for carriers of these gene variants would focus on achieving high plasma ratios of VE/cholesterol or VE/triglycerides to maximize the opportunity of capturing VE from the circulation by the tissues.

### 3.3. Impaired Liver Vitamin E Balance (Storage versus Catabolism)

Liver metabolism, storage, and catabolism of VE are well-characterized processes [5,94]. Tocopherol transport proteins and the cytochromes involved in its catabolism are two important regulatory points of VE metabolism, not only in the liver, but also at the systemic level [130]. Therefore, genetic variants in the genes concerned are associated with phenotypes that modulate the capacity to retain VE in the liver and/or to undertake faster catabolism. It is worth mentioning that the role of metabolic products derived from the catabolism of α-tocopherol is still uncertain. In fact, some studies point toward these products being the ones that would really be behind many of the beneficial effects attributed to VE, such as α-carboxyethyl-6-hydroxychromans (CEHC), which is related to anti-proliferative [131] and anti-atherosclerotic potential [132], and could offer specific protection against inflammation [133] and oxidation [134] (for more details of the effects of VE derived metabolites and human benefits, see the reviews of Wallert et al. (2014) and Schubert et al. (2018) [135,136]). Here, we focus on the main proteins involved in liver storage/handling of VE and the most characterized elements of the catabolism machinery.

Tocopherol transfer protein α (αTTP) is a 32-kDa cytosolic protein encoded by the *TTPA* gene; it is also highly expressed in liver, which binds α-tocopherol with high selectivity and affinity [44]. This protein plays an important role in regulating α-tocopherol storage in liver and its transport between membrane vesicles, facilitating the package of VE in VLDLs, its secretion from hepatocytes, and delivery to extrahepatic tissues [137]. The role of αTTP in VE homeostasis is well documented: in rats, a diet deficient in α-tocopherol induces lower αTTP levels in the liver [138]; mutations in the *TTPA* gene are related to ataxia in humans, which is a neurological dysfunction that includes vitamin E serum deficiency [139]. Furthermore, *TTPA* expression is induced by oxidative stress or hypoxia via the transcription factor cAMP response element-binding (CREB) [130].

Three variants in the promoter of αTTP: 1753C/T (rs1205682), 1410A/T (rs6472071), and 344C/T (rs74684018) have been related with higher transcriptional rates, while the repression of the promotor activity has been found for 980A/T (rs6994076), 945A/G (rs34358293), 675G/A (rs80169698), and 439A/G (rs73684515) substitutions [130]. Accordingly, these SNPs might influence the balance of α-tocopherol between liver and tissues. In the Alpha-Tocopherol, Beta-Carotene Cancer Prevention (ATBC) intervention study, the presence of the T/T genotype of the 980A/T polymorphism was associated with approximately 3% lower serum levels of αtocopherol at baseline in comparison with the other genotypes. Furthermore, a lower response (25%) to VE supplementation was observed in serum α-tocopherol concentration in T/T subjects in comparison with those carrying two copies of the common allele [140].

Associated with the lipophilic properties of VE, other additional binding proteins contribute to its cellular homeostasis. The human α-tocopherol associated protein (hTAP), encoded by *SEC14L2* gene, is ubiquitously expressed [141]; it binds cytosolic α-tocopherol and contributes to its translocation to the nucleus, where it is able to modulate gene expression [142], or to other organelles such as mitochondria, where it contributes to the maintenance of the oxidative balance of membrane lipids, and protects from reactive oxygen species [142,143]. A few polymorphisms in *SEC14L2* have been associated with slightly higher serum concentrations among men carrying the two copies of the variant allele in comparison with the common allele, and a modest serum response to VE supplementation has also been observed [140].

The catabolism of VE is an important, regulated physiological step to avoid the excessive accumulation of VE in liver and tissues. After the control exerted at the absorption step, the metabolism of VE to derivatives seems to be the second main regulatory stage for the well-balanced vitamin E status.

Cytochrome P450 is a superfamily of cytochromes that catalyzes a key -hydroxylation reaction in the pathway of VE catabolism [144] and of other bioactive compounds such as leukotriene-B4 and arachidonic acid [145]. Low affinity forms of VE for α-TTP, such as ɤ-tocopherol, are assumed to be excreted via bile acids or eliminated in the urine after conversion to CEHC in hepatocytes, which takes place faster than with α-tocopherol forms, which are also catabolized to counteract excess VE when the capacity of liver α-TTP is exceeded [38,136]. Cytochromes CYP34A and CYP4F2 appear to be the isoforms that are involved in the oxidation of α-tocopherol and ɤ-tocopherol, respectively [145,146]. In mice, depletion of the functional orthologue of human *CYP4F2*, causes ɤ-tocopherol accumulation in plasma and tissues, and a reduction in the excretion of -oxidation metabolites [147]. An early in silico study identified cytochrome genes *CYP3A4* and *CYP4F2* as highly polymorphic in the context of proteins that are important in VE homeostasis; therefore, they were proposed as candidates to account for individual variability in the steady state of VE status or in response to supplementation [148]. Two SNPs in *CYP4F2* have been further characterized in in vitro systems, showing functional changes in the enzymatic activity of the cytochrome. The rs3093105 involves a T to G nucleotide exchange, leading to a tryptophan to glycine substitution at amino acid position 12 (W12G), with this polymorphism showing increased enzymatic activity in hydroxylating both tocopherols and tocotrienols. Dissimilar rs2108622 entails a G to A nucleotide exchange causing the substitution of avaline for methionine (V433M). This variant is associated with decreased baseline α-tocopherol levels (β = 0.51) [149], and has been related with decreased enzymatic activity for tocopherols, but does not affect the hydroxylation of tocotrienols [150]. Although the differential performance in the catabolism of VE and its derivatives associated to cytochrome P450 might explain inconsistent findings after supplementation trials, some data from human intervention studies are not so consistent, and point toward polymorphisms on *CYP4F2* possibly playing a moderate role in affecting the pharmacokinetics of VE [149,151].

### 3.4. Factors Compromising the Neutralization of Situations of Oxidative Stress

Beyond the principal genetic variants that are able to modulate VE bioavailability and its hepatic balance, there is a core of evidence stating that biological mechanisms dealing with oxidative stress conditions, low-grade inflammation as observed in obesity, and other factors contributing to the development of the atherogenic process could be influenced by VE status. The hypothesis behind this rationale is mainly based on the antioxidant potential of VE, which would counteract some of the adverse effects of oxidative stress that are characteristic of cardiovascular diseases, such as obesity and atherosclerosis. However, taking into account recent findings, this may not be exclusively associated with its role as an antioxidant, but rather related to other emerging additional properties that are uncharacterized as yet [5]. Below, there are a few representative examples that outline the complex role of VE in metabolism and the modulatory influence of genotype. 

VE is an antioxidant with a known immunoregulatory potential; it is able to boost host protection against bacterial infection [152,153,154]. Specific studies have nicely shown the mediation of genetic factors on immune responses after VE supplementation in healthy humans, such as those found in the production of cytokines and/or related with the cytokine genes *TNF*, *IL-6*, and *IL-1B*. Carriers of the A allele of the -308G>A polymorphism in the *TNFα* gene (rs1800629) has been associated with the increased production of TNFα [155], and this expression is modulated by VE status. Thus, immunity cells from healthy individuals, isolated after one year of oral α-tocopherol supplementation (182 mg/day), and then stimulated with lipopolysaccharide (LPS), have shown that genotypes A/G or A/A produced lower levels of TNFα in response to LPS, suggesting that the anti-inflammatory effect of VE is specifically targeted in those individuals who are genetically predisposed to show higher inflammation [156]. In a similar experimental design to study the influence of variants on the inflammatory response after VE supplementation, carriers of the rare allele (A) of SNP rs361525 (TNF*α* 238G/A) were identified as those showing the decreased production of TNFα; however, this study did not reproduce the above-mentioned effects observed for rs1800629 [157]. The latter study also observed that α-tocopherol supplementation in healthy individuals influences the production of inflammatory cytokines in a genotype-dependent manner. Thus, the presence of the polymorphism rs1695 in glutathione S-transferase gene (*GSTP1*), which codifies an important protein for detoxification, influences IL-6 and the polymorphism rs1800896 located in IL10 gene promoter, and modulates the production of IL-1β.

Enzymes involved in the detoxification pathway are also relevant in the action of VE. For instance, genetic variant rs2301241, which is also called 793T > C and is located in the promoter of the gene coding for Thioredoxin (*TXN*), is related to oxidative stress [158], and modulates the transcriptional activity of the gene [159]. Polymorphisms in *TXN* may affect adiposity, depending on VE status. Therefore, carriers of allele G for rs4135168 and of the genotype T/T for rs2301241, both in gene *TXN*, showed larger waist circumference in conjunction with low dietary intake of VE (<nine mg/day); the same trend was observed in G/G carriers for rs740603 in the catechol-O-methyltransferase (*COMT*) gene in comparison with other genotypes [159]. Interestingly, these relationships are not sustained among subjects that regularly have higher intakes of VE (≥nine mg/day).

Finally, Sirtuin 1 is a member of a protein family of NAD^+^-dependent histone deacetylases involved in various physiological processes such as energy metabolism, and it also protects cells against oxidative stress [160,161]. The decreased expression of *SIRT1* is related to obesity [162] and genetic variants located in the *SIRT1* gene have been associated with body mass index (BMI) and obesity risk; specifically, a lower BMI has been observed in carriers of the minor alleles of rs7895833 (G) and rs1467568 (A) [161]. Furthermore, a gene–nutrient–phenotype relationship has been found among dietary vitamin E and genetic variants in *SIRT1* in relation to BMI. Unexpectedly, elevated risk to having a greater BMI was related to a higher copy number of the most common haplotype (alleles A of rs7895833, G of rs1467568, and G of rs497849) among subjects with low VE intake [161], suggesting the capacity of VE to modify the association between SIRT1 variants and obesity.

## 4. Concluding Remarks

Vitamin E has a recognized leading role as a contributor to the protection of cell constituents from oxidative damage, although the health benefits of VE probably go far beyond its involvement as a radical scavenger. A full understanding of the metabolic targets of VE or its derivatives, as well as their mechanism of action, is still lacking. Interestingly, no specific transport protein has been described for VE, and its absorption, handling, and distribution share common pathways associated with the transmembrane transport of cholesterol and lipoproteins. Thus, critical pathways in obesity and metabolic syndrome may be under the influence of VE, and variation in the genes involved in VE metabolism might be of relevance, determining both VE supply and lipid metabolism performance. Nonetheless, more research is needed to gain better knowledge of the nutrigenetic influence of VE; in specific cases, appropriate strategies could be defined to counteract the impaired bioavailability of VE, even in the case of high plasma VE. However, under current knowledge, it seems more difficult to give adjusted VE dietary recommendations to tackle impaired liver VE balance or efficiently neutralize situations of oxidative stress. The metabolic effects of VE and the influence of genetic variants remains a challenge for precision nutrition in obesity and associated disturbances.
